# Recent Therapeutics

**Published:** 1890-09-13

**Authors:** 


### THERAPEUTICS.

### RECENT THERAPEUTICS.

PHENACETIN FOR ACUTE RHEUMATISM—PILOCARPINE FOR CHRONIC RHEUMATISM—HYDROFLUORIC ACID INHALATIONS FOR PHTHISIS — EUPHORBIA PILULIFERA FOR ASTHMA — ACTION OF CODEINE — TOXIC EFFECTS OF COCAINE — HYDROCHINONE — RHUS AROMATICA FOR ENURESIS—CUBEBIC ACID—NITRATE OF SILVER—CASCARA AMARGO—OREXIN.

Acute rheumatism, whether of a general or specific character, has been treated in very many perfectly different manners, yet no particular treatment has become generally adopted. Recently phenacetin in large doses has been recommended with renewed vigour by Dr. Refat, in the *Bulletin Général de Therapeutique*. He has given forty-five, ninety, and even one hundred grains in divided doses during the twenty-four hours. The average duration of treatment was seventeen days. The author states that phenacetin does not excite toxic effects until 15 grains for every pound of body weight have been administered. Nevertheless, there are peculiar idiosyncracies and constitutions. And we fear that the profuse sweating, cyanosis, and uræmic poisoning, which have been recorded after the use of phenacetin, might be produced in some persons long before 15 grains to the pound had been taken. Pilocarpine has been several times recommended for chronic articular rheumatism, the most recent suggestion of the kind being by Dr. Hochhalt, of Berlin, in June last. The author believes the pilocarpine will to a very great extent relieve chronic articular rheumatism, provided the case is not of very long standing. The action of pilocarpine is to increase the activity of the salivary and perspiratory glands, by which probably the results of tissue metamorphosis are eliminated. Any means by which chronic articular rheumatism may be relieved would be hailed as a blessing by thousands. It may, therefore, be well to bear in mind Dr. Hochhalt's remarks on pilocarpine.

Inhalations of various kinds have been frequently used for phthisis, and some time ago we commented on the treatment by inhalation of super-heated air. One of the last recommendations, by Dr. Garcin, of Paris, is inhalation of hydrofluoric acid. Hydrofluoric acid is a microbicide of the most powerful nature. Dr. Garcin states that in the great majority of cases inhalations of this acid considerably improve the general state, and permit the patients to resume their ordinary occupations. The cough is lessened, the sweating stopped, difficulty of breathing relieved, while appetite and strength return.

Dr. R. Workman, of Keyser, W.V.A., in a late number of the *Philadelphia Therapeutical Gazette*, recommends a fluid extractof *Euphorbia piliulifera* for hay asthma and allied complaints. He has notes of twenty-seven cases in which the drug was given. In thirteen cases of hay asthma prompt relief was obtained in nine. The sneezing and discharge were diminished or stopped, and the sensation of congestion relieved. In nine cases of coryza good results were obtained, in six the rhinal flow ceasing soon after the administration of the medicine. In five cases of asthma marked relief was obtained. The dose of the fluid extract is from thirty to sixty minims every four hours. But with reference to hay asthma or hay fever, it has been pointed out that the symptoms are peversions or exaggerations of the normal nasal reflexes, *plus* signs of nasal irritation. Examination generally discloses local hyperæmia, and many medical men place most faith in local medication. Dr. Scanes Spicer has devised medicated nasal cylinders, which, while permitting free nasal respiration, soothe the affected tract. As a general rule, however, change of air is more likely to benefit hay fever or hay asthma than any kind of medicine. Unfortunately we cannot always say what change will be beneficial. We may, however, generally advise a radical change, such as from inland to the sea, or the reverse, with confidence.

An elaborate paper on the action of codeine has been lately published by Dr. Loewenmeyer, of Berlin, who has used the drug extensively. The author states that codeine is analogous in chemical composition and activity to morphine, but it possesses a less intense narcotic action, and is absolutely free from unfavourable influences. Codeine is recommended in various conditions of the pelvic and abdominal organs accompanied by pain, such as gastralgia, colic, and pain located in the pelvis, without any recognisable cause. So also in cases where the pain is evidently dependent upon marked anatomical alterations, such as gastric ulcer, or cancer of the liver, or intestines. But it appears that in cases in which the pain is paroxysmal, codeine will afford but little relief. It therefore failed in cases of biliary and renal colic. In diseases of the thorax and respiratory tract, codeine is especially useful. In phthisis morphine is not to be compared with codeine as capable of removing pain in the side, feeling of pressure in the chest, dyspnœa, and distressing painful cough. Heart disease is said to be no contraindication to its use, and even in cases of marked fatty heart it may be used with success. The dose is from $\frac{1}{3}$ to $\frac{3}{4}$ of a grain given in powdered form, in capsules, or in solution sweetened with syrup to disguise the unpleasant taste. The editor of the *Philadelphia Therapeutical Gazette*, in commenting on Dr. Loewenmeyer's paper as above, remarks that unfortunately codeine costs nearly three times as much as morphine, and nearly three times as large doses are required, so that its use is "therefore a somewhat costly experiment." And it is added that as in the case of many other drugs, the usefulness of codeine greatly depends upon its mode of administration, "and it should be remembered that codeine while soluble in alcohol and ether is almost insoluble in water . . . . Where it is desired that codeine should enter into any watery preparation, the hydrochlorate, sulphate, or phosphate should be chosen on account of their greater solubility, while the phosphate is best for subcutaneous application, as it dissolves in four parts of water, and rarely produces local irritation."

In the same journal we find an article on the toxic effects of cocaine. The writer remarks that "it is a striking fact that the use of cocaine in the eye seems to be most free from unpleasant results, while application to the ear, nose, or throat has been followed by alarming symptoms." In the *New York Medical Record* for June last Dr. Isidor Gluck states that a combination of cocaine with phenol abolishes all risk of unpleasant consequences. The formula is two drops of phenol dissolved in one drachm of distilled water shaking until solution is perfect, and then adding ten grains of cocaine hydrochlorate. The advantages claimed are that phenol is also a local anæsthetic; that phenol coagulates albumen and combines with fats forming a very superficial eschar, which prevents the rapid absorption of cocaine and hence its toxic effects; that phenol is also destructive of bacteria and prevents decomposition.

The now extensive list of antipyretic medicines is again increased by the addition of hydrochinone. It is some years since this substance was first credited with antithermic properties, but such properties were not regarded as sufficient to entitle hydrochinone to much attention. According, however, to recent investigations by Dr. Traversa, it would appear that hydrochinone is a decided antipyretic. He states that the drug reduces the temperature both by dissipation of heat, and by preventing the production of heat, by retarding the chemical processes and oxidation which occur in the organism. The antipyretic action is not ordinarily accompanied by any disagreeable symptoms.

In the "Retrospect" for August 2nd last, we remarked on nocturnal incontinence of children, with reference to a plan of treatment advocated by Dr. W. A. Briggs, of Sacramento. This consists in the employment of electricity, a sound being introduced into the bladder. We then remarked that manipulation with instruments could rarely be required, and might often be very injurious. We now find Dr. W. Powell, of New York, advocating the use of *rhus aromatica* for the enuresis of children. The following is the formula used: Ext. rhois aromat., ʒiii.; elix. aromatic, ℥ss.; aquæ cinnam. q. s. ad., ℥iii.; half a-teaspoonful to be increased to one teaspoonful four times a-day after eating. Dr. Powell, however, remarks that much stress should be laid upon diet and general hygiene, giving much the same advice as contained in our article of August 2nd. Dr. Powell adds that drink should be restricted, and no fluid should be allowed after four or five o'clock in the afternoon. If the patient shows any rheumatic symptom, none or very little meat should be allowed, which tends to acidifying the urine, irritating the bladder, and provoking expulsion.

Instead of cubebs for the treatment of gonorrhœa, Bernatzik has recently recommended cubebic acid (*Jour. de Méd. de Paris*, July, '90). He states that the antiblennorrhagic action of cubebs is principally due to the presence of cubebic acid, and that the administration of cubebic acid alone will produce more reliable results and a more certain cure. Cubebic acid is a whitish substance, resembling wax, which becomes discoloured brown when exposed to the air, and is very soluble in ether and alcohol. The maximum dose is fifteen grams, but not more than seventy-five grams should be given in twenty-four hours. Dr. Friedheim, of Breslau, however, regards injection of nitrate of silver as the best treatment. The strength is 1 in 4,000 to 1 in 2,000. The injection, used from four to six times daily, diminishes the gonococci in a remarkable manner, and after a few days they disappear altogether. The number of injections is then reduced gradually to one daily. As regards the danger of complications with which the use of nitrate of silver has been credited, the author remarks that it was found this mode of treatment was the best preventive, there being fewer complications under the nitrate of silver method than with other remedies. Dr. Malecot, of Paris, also regards the prejudice against the use of nitrate of silver as unfounded.

Dr. C. H. Haines, of Auckland, N.Z., believes that cascara amarga is almost specific for the ulcerations of tertiary syphilis. Several cases of immediate and marked improvement are detailed by Dr. Haines. But for secondary symptoms he has not seen good follow its exhibition. In cases where mercury is contraindicated, and where the depressing effects of iodides are dreaded, cascara amarga is most beneficial. The form of the drug used is a fluid extract, and if thought desirable it may be combined with iodide of potassium. The fluid extract used by Dr. Haines was prepared by Messrs. Parke, Davis, and Co., of Philadelphia.

The editor of the *Philadelphia Therapeutical Gazette* has an annotation headed, "The Last of Orexin," which it is said may now be relegated to the obscurity it seems to deserve. Dr. George Mullen, in common with several other observers, has found orexin to be absolutely inert, notwithstanding the remarkable powers of stimulating the appetite, &c., which have been credited to it. We have no doubt that before very long the heading as above may be applied to a number of now vaunted drugs flourishing under false attributes, but soon not to be heard of for some generations at least, until again revived into a temporary celebrity.

### LOCAL ANÆSTHESIA BY CARBONIC ACID GAS.

Dr. Voituriez recommends what he considers a new and simple means of producing local and superficial anæsthesia, by projecting on the site of the intended operation, the contents of two or three syphon bottles of soda or seltzer water from a distance of three or four inches. The anæsthesia lasts for about five minutes, but if requisite the spraying can be repeated. This method should be restricted to minor operations on the limbs, as it appears to be undesirable to apply it to the head or neck. Sensibility returns slowly, so that a single application will suffice for operations occupying even ten or fifteen minutes. It may be found useful when ordinary ether-sprays are not at hand.

### VINOLIA.

Every medical man has been, during the the past year favoured, we might say, pestered, with prospectuses and samples of the preparations bearing this name, which, we presume has been suggested by the scriptural expression of pouring wine and oil into wounds. The soap is certainly a most elegant and admirable article for the toilet, being entirely void of free alkali, and making an excellent tooth-paste for this reason. But it is well to state that the cream or vinolia itself, and the vinolia powder are totally distinct preparations, having nothing whatever in common. The former is Lanolin, with phenol, salicylic acid, and thymol; the latter is simply finely powdered and perfumed Boric acid. N.B. Please compare the prices.

